# Telemedicine and Depression Screening After the Start of the COVID-19 Pandemic

**DOI:** 10.1001/jamanetworkopen.2023.55830

**Published:** 2024-02-12

**Authors:** Maria E. Garcia, John Neuhaus, Jennifer Livaudais-Toman, Mitchell D. Feldman, Lisa Ochoa-Frongia, Elaine Khoong, Leah S. Karliner

**Affiliations:** 1 Mt Zion Division of General Internal Medicine, Department of Medicine, University of California, San Francisco; 2 Department of Epidemiology and Biostatistics, University of California, San Francisco; 3 Zuckerberg San Francisco General Hospital Division of General Internal Medicine, Department of Medicine, University of California, San Francisco

## Abstract

This cohort study investigates the probability of depression screening by visit type and by patient demographic characteristics in a large health system during the early COVID-19 pandemic.

## Introduction

Depressive symptoms increased during the COVID-19 pandemic.^1,2^ While use of telemedicine also increased during this time, little is known about depression screening during telemedicine encounters in primary care. We investigated the probability of depression screening by visit type and patient characteristics in a large health system early during the COVID-19 pandemic.

## Methods

This cohort study assessed adult depression screening in 6 University of California, San Francisco, primary care practices from June 1, 2020, to September 30, 2021, using electronic health record data. Depression screening was defined as completion of the Patient Health Questionnaire 2 (PHQ-2) during an eligible visit. Visits were excluded if patients had been screened in the preceding 12 months; had a baseline diagnosis of depression, bipolar disorder, schizophrenia, schizoaffective disorder, or dementia; or had a new diagnosis of depression during the study. Visits were categorized as in person, video, or telephone using billing information. Additional covariates included self-reported race and ethnicity, preferred language, sex, age, baseline Elixhauser comorbidity count, and primary insurance type, which have been associated with depression screening disparities.

We calculated monthly depression screening rates by visit type. We then evaluated the odds of screening at an eligible visit using a mixed-effects logistic regression model with random patient effects to accommodate multiple visits per patient, adjusting for primary care site and month of visit.

We used Stata, version 16.1^3^ for data analysis from December 2021 to June 2023; statistical significance was set at *P* < .05. The study was approved by the University of California, San Francisco, Institutional Review Board with a waiver of consent owing to use of routine clinical care data. We followed the STROBE reporting guideline.

## Results

There were 57 301 eligible visits for 37 250 unique patients during the study period (60% female and 40% male; mean [SD] age, 52.3 [17.9] years), including 8% African American or Black, 0.3% American Indian or Alaska Native, 30% Asian, 10% Hispanic or Latinx, 1% Native Hawaiian or Other Pacific Islander, 42% White, and 8% other or unknown. Language preference was non-English in 7%. The Figure presents depression screening during eligible visits by visit type and month.

**Figure.**
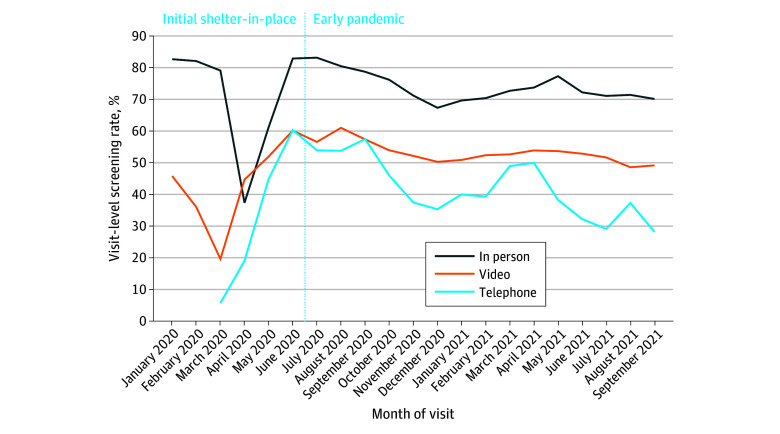
Monthly Depression Screening Rates at Eligible Visits by Visit Type From June 2020 to September 2021 The prestudy period (January to May 2020) demonstrates baseline screening rates.

Patients had lower odds of being screened in video and telephone visits compared with in-person visits (Table). While there were no differences in screening by sex or race and ethnicity, patients who preferred Chinese, Spanish, or other languages had lower odds of screening than patients who preferred English (Table). Patients older than 75 years had lower odds of being screened than patients aged 18 to 30 years. Patients with Medicaid were less likely to be screened than those with private insurance; for each additional comorbidity, the odds of being screened decreased.

**Table.** AORs From a Logistic Regression Model of Depression Screening During Eligible Visits Early in the COVID-19 Pandemic^a^

Characteristic	Screening at visit, AOR (95% CI)
Visit type	
Video	0.28 (0.27-0.30)
Telephone	0.24 (0.20-0.27)
In person	1 [Reference]
Sex	
Men	1.04 (0.99-1.10)
Women	1 [Reference]
Language preference	
Chinese^b^	0.70 (0.59-0.82)
Spanish	0.59 (0.43-0.80)
Other	0.69 (0.57-0.82)
English	1 [Reference]
Race and ethnicity	
African American or Black	0.93 (0.83-1.04)
American Indian or Alaska Native	0.76 (0.47-1.25)
Asian	1.06 (0.99-1.14)
Hispanic or Latinx	0.98 (0.89-1.08)
Native Hawaiian or Other Pacific Islander	0.94 (0.75-1.18)
White	1 [Reference]
Other or unknown	0.96 (0.87-1.06)
Age category, y	
18-30	1 [Reference]
31-44	0.99 (0.91-1.08)
45-54	0.99 (0.90-1.09)
55-64	1.03 (0.93-1.14)
65-74	1.07 (0.94-1.22)
≥75	0.79 (0.68-0.92)
No. of comorbidities (continuous)	0.93 (0.91-0.96)
Health insurance	
Medicare	0.97 (0.88-1.08)
Medicaid	0.80 (0.73-0.88)
Other^c^	0.93 (0.72-1.20)
Private	1 [Reference]

Abbreviation: AOR, adjusted odds ratio.

^a^ Study period includes June 1, 2020, to September 30, 2021, and 57 301 visits. Model accounts for multiple visits by patient and is adjusted for month of visit.

^b^ Includes patients who spoke Mandarin and Cantonese as well as other Chinese dialects.

^c^ Includes self-pay, Veterans Affairs, or worker’s compensation.

## Discussion

In this cohort study conducted within a large health system, depression screening rates during video and telephone visits plateaued quickly and remained lower compared with in-person visits in the early period of the COVID-19 pandemic. Depression screening workflows and protocols were initially developed for in-person visits.^4^ Due to the abrupt transition to telemedicine early in the pandemic and the urgency of the pandemic response, team-based workflows for depression screening lagged for telemedicine encounters. Many primary care practices offer regular telemedicine visits^5^; to avoid missing individuals with depressive symptoms, primary care must fully incorporate screening into telemedicine visits. Additional systems solutions that remove reliance on support staff, such as sending PHQ-9s through the patient portal prior to visits, may have a high yield for some populations.

Although disparities in screening had been eliminated before the study period for older adults and patients with non–English language preference, multiple comorbidities, and Medicaid,^4^ these disparities recurred and persisted during the study period for all visit types. Health systems must be vigilant and proactive in addressing emerging disparities by race and ethnicity, language, or age, particularly in the telemedicine setting. Study limitations include the single site, inability to capture patient refusal of screening, and visit-level vs individual-level analysis.

## Notes

Supplement.
Data Sharing Statement
