# Supplementary material for: Telemedicine and Depression Screening After the Start of the COVID-19 Pandemic
Source: JAMA Netw Open. 2024 Feb 12;7(2):e2355830. doi: 10.1001/jamanetworkopen.2023.55830 (PMC10862145; doi:10.1001/jamanetworkopen.2023.55830)
Supplement: Supplement. — Data Sharing Statement [file jamanetwopen-e2355830-s001.pdf]

Data Sharing Statement

Garcia. Telemedicine and Depression Screening After the Start of the COVID-19 Pandemic. *JAMA Netw Open*. Published February 12, 2024. doi:10.1001/jamanetworkopen.2023.55830

Data
Data available: No
